# Pandemic fatigue among young doctors during the COVID-19 pandemic: The mediating role of resilience

**DOI:** 10.1192/j.eurpsy.2023.1245

**Published:** 2023-07-19

**Authors:** N. Kotti, R. Masmoudi, I. Sellami, A. Hrairi, K. Jmal Hammami, M. L. Masmoudi, J. Masmoudi, M. Hajjaji

**Affiliations:** 1 Occupational medecine; 2 Psychiatry A department, Hedi Chaker Hospital, Sfax, Tunisia

## Abstract

**Introduction:**

The ongoing pandemic due to coronavirus disease (COVID-19) is not only causing casualties amongst patients but is also putting an enormous strain on healthcare workers worldwide, especially those in frontline of the COVID-19.

**Objectives:**

This study examined the influence of pandemic fatigue on physicians’ mental health, with resilience as a mediator.

**Methods:**

This was a descriptive, cross-sectional study involving frontline young doctors at two university hospitals of Sfax, Tunisia. The Pandemic Fatigue Questionnaire, Brief resilience scale, Maslach Burnout Inventory and Satisfaction on call duty scale were used to collect data through an online survey. The survey was carried out through an anonymous questionnaire using Google Forms. Collected data was treated on SPSS program to make all the statistical analysis. The level of statistical significance was set at p<0.05.

**Results:**

A total of 261 young doctors responded to the online survey. The mean pandemic fatigue score was 25.09 (out of 50). Terminal years of residency experience (β=0.171, p=0.005), being vaccinated (β=0.129, p=0.032) and staff inadequacy (β=0.205, p=0.001) were associated with elevated score of pandemic fatigue. Resilience partially mediated the relationships between (a) pandemic fatigue and different dimensions of burnout (emotional exhaustion (β=0.337, p<0.0001), depersonalization (β=0.311, p<0.0001) and personal accomplishment (β= =0.185, p=0.004) and (b) pandemic fatigue and satisfaction on call duty (β=-0.137, p=0.03).

**Conclusions:**

Resilience reduces the effects of pandemic fatigue on young doctors’ mental health. Implementing resilience-promoting measures is essential to support physicians’ mental health and foster their well-being therefore improves the quality of care provided.

**Disclosure of Interest:**

None Declared

